# Effects of covid-19 work circumstances on mental health

**DOI:** 10.1192/j.eurpsy.2023.1726

**Published:** 2023-07-19

**Authors:** W. Ayed, S. Chebbi, M. Mosbeh, A. Ayadi, S. Ayari, I. Magroun

**Affiliations:** Occupational health departement, University of Tunis El Manar - faculty of Medicine of Tunis, Ariana, Tunisia

## Abstract

**Introduction:**

Post-traumatic stress disorder (PTSD) was described among patients with COVID-19, health professionals (HP), and the population at large. HP were in the front-line managing this pandemic which put them at a higher risk to develop such trouble.

**Objectives:**

The aim of our study was to evaluate the effect of work circumstances on the mental health of HP.

**Methods:**

Cross-sectional descriptive study was carried out. It included HP at Abdurrahman Mami Hospital who had a positive RT-PCR test of SARS-CoV-2 on a nasopharyngeal swab over the 10-month period from January to October 2021. Data collection was performed three months after the resumption using the PCL-5 questionnaire .

**Results:**

Seventy six HP was included in our study. PTSD incidence was 30%. Age average was 41 ± 9 years. Women represented 84%. Seventy eight percent of the HP were married and 71% were living with their children. The average number of persons in the family was 4 ± 1. Intensive care unit was the department of origin for 17% of the HP, the laboratory in 8% of the cases, the emergency room in 3% and the Covid-19 hospitalization services in 24%. The most affected occupational category was nurses (39%), laboratory technicians (14%), and physicians (8%). General difficulties with tasks usually performed was found in 35% of HP suffering from PTSD (p=0.012). It appears that limiting the time spent at work had a protective role for PTSD with an OR of 0.25 (p=0.002).

**Conclusions:**

Handling COVID-19 health issues was the concern of all medical departments. This study highlights the impact of work circumstances on the mental health of workers. PTSD was prevalent among HP according to our study. PTSD may have an impact on work ability, which should be further explored by other studies. Other psychiatric disorders should also be investigated.

**Disclosure of Interest:**

None Declared

